# Supplementary material for: Mechanisms governing the pioneering and redistribution capabilities of the non-classical pioneer PU.1
Source: Nat Commun. 2020 Jan 21;11:402. doi: 10.1038/s41467-019-13960-2 (PMC6972792; doi:10.1038/s41467-019-13960-2)
Supplement: Supplementary file 7 — Source data [file 41467_2019_13960_MOESM7_ESM.zip › Source_Data/FigureS5/FigureS5A_MotifsCommonPeaks/homerResults/motif3.similar.html]

motif3

## Information for motif3

C
G
A
T
T
C
A
G
G
A
C
T
T
A
C
G
A
T
C
G
A
G
C
T
G
A
C
T
C
G
A
T
G
T
A
C
  
Reverse Opposite:  

C
A
T
G
G
C
T
A
C
T
G
A
T
C
G
A
A
T
G
C
A
T
G
C
C
T
G
A
A
G
T
C
G
C
T
A
  

|  |  |
| --- | --- |
| p-value: | 1e-590 |
| log p-value: | -1.360e+03 |
| Information Content per bp: | 1.707 |
| Number of Target Sequences with motif | 3487.0 |
| Percentage of Target Sequences with motif | 39.24% |
| Number of Background Sequences with motif | 5189.3 |
| Percentage of Background Sequences with motif | 13.65% |
| Average Position of motif in Targets | 103.1 +/- 48.9bp |
| Average Position of motif in Background | 98.7 +/- 64.5bp |
| Strand Bias (log2 ratio + to - strand density) | 0.0 |
| Multiplicity (# of sites on avg that occur together) | 1.24 |
| Motif File: | file (matrix) reverse opposite |

### Similar de novo motifs found

|  |  |  |  |  |  |  |  |
| --- | --- | --- | --- | --- | --- | --- | --- |
| Rank | Match Score | Redundant Motif | P-value | log P-value | % of Targets | % of Background | Motif file |
| 1 | 0.965 | C G A T A C T G G A C T A C T G T A C G A C G T G C A T C G T A T C A G A G T C | 1e-584 | -1346.534094 | 31.58% | 8.90% | motif file (matrix) |
| 2 | 0.977 | C A T G C A T G C A T G A C T G G A T C C G A T A T C G C G A T C A T G T A C G A G C T A G C T C G A T | 1e-569 | -1310.813139 | 32.56% | 9.70% | motif file (matrix) |
| 3 | 0.984 | T G C A C T A G C A T G G C T A T C G A T C G A A G T C A G T C C T G A T A G C G C T A | 1e-559 | -1288.243939 | 36.02% | 12.03% | motif file (matrix) |
| 4 | 0.993 | C G A T A C T G G A C T A C T G A C T G A G C T G A C T C G T A | 1e-541 | -1245.946244 | 33.73% | 10.85% | motif file (matrix) |
| 5 | 0.970 | T G C A T C A G T A C G G C T A C T G A T C G A A G T C A G T C C G T A A G T C G C T A C T A G | 1e-520 | -1199.204381 | 29.79% | 8.74% | motif file (matrix) |
| 6 | 0.982 | A C G T A C T G A G C T A C T G A C T G A C G T G A C T | 1e-457 | -1054.114338 | 29.51% | 9.53% | motif file (matrix) |
| 7 | 0.947 | G A C T A G C T A C T G G A C T A T C G A C T G A G C T A C G T A C G T A G T C | 1e-412 | -949.484221 | 28.20% | 9.46% | motif file (matrix) |
| 8 | 0.955 | T C G A T C G A A G T C T G A C C G T A G T A C G C T A | 1e-406 | -936.964636 | 32.29% | 12.23% | motif file (matrix) |
| 9 | 0.845 | A C T G A G T C A T C G A C T G A C G T C G A T C G T A | 1e-353 | -813.140429 | 38.52% | 17.93% | motif file (matrix) |
| 10 | 0.959 | G C T A T C G A T C G A A G T C A G T C G T C A A T G C G T C A T C A G T G C A T A G C G T C A G T C A T A G C | 1e-351 | -809.869329 | 19.18% | 5.09% | motif file (matrix) |
| 11 | 0.895 | T C G A A G T C A G T C C G T A A G T C C G T A A G C T | 1e-332 | -766.230479 | 57.45% | 34.75% | motif file (matrix) |
| 12 | 0.853 | T G A C A G T C G A C T G C T A C G T A A G T C A G T C C T G A T G A C | 1e-301 | -693.472189 | 32.29% | 14.52% | motif file (matrix) |
| 13 | 0.853 | T C G A T C G A A G T C A G T C C T G A A G T C T G C A G C T A | 1e-188 | -433.524097 | 13.59% | 4.44% | motif file (matrix) |
| 14 | 0.809 | T A C G T A C G G T A C A G T C C G T A A G T C T C G A | 1e-158 | -365.963306 | 40.54% | 25.84% | motif file (matrix) |
| 15 | 0.865 | C A G T G A T C A C G T A C T G A C G T A C T G A C T G A G C T T C G A C T G A T C A G C T G A | 1e-134 | -310.616647 | 10.85% | 3.79% | motif file (matrix) |
| 16 | 0.836 | G A T C A G T C G A C T A G C T T C G A A G T C A G T C C G T A G A T C G C T A C T G A | 1e-106 | -246.150754 | 8.88% | 3.15% | motif file (matrix) |
| 17 | 0.781 | C T G A C T G A A G T C A T G C A C T G G T A C G C T A T C G A | 1e-71 | -165.626485 | 11.56% | 5.83% | motif file (matrix) |
| 18 | 0.816 | G T C A C T G A A G T C A G T C A T G C C G T A A G T C C G T A | 1e-68 | -157.832943 | 10.87% | 5.45% | motif file (matrix) |
| 19 | 0.778 | A C G T T A C G A C T G A C G T A C T G A C T G A C G T A C G T | 1e-28 | -65.709002 | 3.43% | 1.50% | motif file (matrix) |
| 20 | 0.802 | A C G T A C T G C G T A A C T G A C T G A C G T A C G T | 1e-19 | -45.999750 | 5.45% | 3.28% | motif file (matrix) |
